# Making waves in structure-based ligand discovery

**DOI:** 10.1063/4.0000948

**Published:** 2025-10-27

**Authors:** Justin T Seffernick, Marcus Fischer

**Affiliations:** St. Jude, Memphis, TN, USA

## Abstract

Water networks contribute to ligand binding but are often ignored during ligand discovery and design. With 95% of all PDB structures collected under cryogenic conditions, the utility of crystallographic waters is currently limited, since water networks change with temperature. To address this shortcoming, we developed ColdBrew to link the impact of temperature on water to displaceability and energy. First, we predicted the probability of a cryogenic water molecule to appear at room temperature using a random forest machine learning approach and a curated dataset of 242 temperature-matched structure pairs. Then, to link ColdBrew probabilities to water displaceability, we analyzed over 1 million water molecules in ligand-bound cryogenic structures. We show that our method can distinguish ligand-displaceable waters from conserved binding site waters. Finally, using inhomogeneous solvation theory we link probabilities from our model to solvent energetics. A case study demonstrates how waters with high ColdBrew probabilities were empirically avoided, with some notable exceptions. Overall, ColdBrew provides a simple, scalable roadmap to assess the utility of water molecules for ligand discovery directly from cryogenic crystal structures.

